# Glutamine metabolic stress induces SLC25A6-dependent mitofission via MIC60–MIC19 complex disassembly in colorectal cancer

**DOI:** 10.1038/s41419-026-08754-6

**Published:** 2026-04-23

**Authors:** Yinong Wang, Bingzhi Wang, Yu Liu, Cheng Zhou, Junhu Yuan, Fanyu Zhang, Ling Ma, Yiming Ma, Hongying Wang

**Affiliations:** 1 State Key Laboratory of Molecular Oncology, National Cancer Center/National Clinical Research Center for Cancer/Cancer Hospital, Chinese Academy of Medical Sciences and Peking Union Medical College, Beijing, China; 2 Department of Pathology, National Cancer Center/National Clinical Research Center for Cancer/Cancer Hospital, Chinese Academy of Medical Sciences and Peking Union Medical College, Beijing, China; 3 Department of Colorectal Surgery, National Cancer Center/National Clinical Research Center for Cancer/ Cancer Hospital, Chinese Academy of Medical Sciences and Peking Union Medical College, Beijing, China

**Keywords:** Colon cancer, Tumour-suppressor proteins

## Abstract

Glutamine addiction is a key metabolic vulnerability in cancer. However, the mechanisms governing the limited efficacy of glutamine metabolism inhibitor (GMI) monotherapy require further investigation. Via single-cell monitoring using a caspase-3 activity indicator, we identified SLC25A6 as a key mediator of GMI-induced apoptosis in colorectal cancer cells. SLC25A6 overexpression enhanced apoptosis both in vitro and in vivo. SLC25A6 promoted mitochondrial fragmentation and dysfunction and upregulated the expression of mitochondrial fission markers. Notably, mitofission inhibitors largely abolished SLC25A6-related mitochondrial dysfunction and intrinsic apoptosis. Mechanistically, SLC25A6 directly interacted with MIC60, competitively inhibiting MIC19 binding; both MIC60 and MIC19 are key components of the mitochondrial contact site and cristae organizing system (MICOS). The SLC25A6 T126A mutant failed to bind MIC60 and lost its ability to destabilize the MICOS complex and facilitate mitofission. Upregulation of SLC25A6 expression induced by the glutaminase inhibitor CB-839 sensitized cancer cells to the Bcl-2 inhibitor ABT-199. Combined CB-839 and ABT-199 treatment showed strong synergistic antitumor effects in colorectal cancer xenograft models. Our findings reveal a novel function of SLC25A6 that links metabolic stress to mitochondrial apoptosis via disruption of the MICOS complex. Combination treatments with mitochondrial apoptotic inducers represent a promising avenue for maximizing the efficacy of GMIs in cancer treatment.

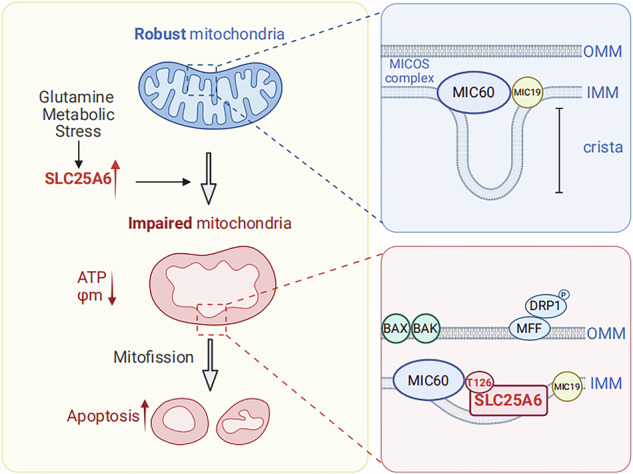

## Introduction

High nutrient demand during tumor progression presents a therapeutic opportunity for cancer treatment. Glutamine addiction is a key metabolic feature of cancer [1]. As a key amino acid that supports energy production via the mitochondrial tricarboxylic acid (TCA) cycle, glutamine serves as a precursor for nucleotides and lipids and maintains the redox balance [2, 3]. To sustain glutamine dependence, cancer cells boost glutamine uptake and upregulate the expression of glutaminase, which converts glutamine to glutamate in the mitochondria and replenishes the TCA cycle. Targeting this metabolic vulnerability via the pharmacological inhibition of glutamine uptake or glutaminase (e.g., CB-839) can effectively suppress tumor growth [4]. However, clinical trials across multiple cancer types have revealed limited efficacy for monotherapies [5].

Glutamine is the predominant fuel for mitochondria, which play key roles in tumorigenesis. Via their participation in the TCA cycle and oxidative phosphorylation, mitochondria serve as core bioenergetic, biosynthetic, and signaling organelles. Multilevel interactions between cellular signaling and mitochondrial function support tumorigenesis and enable cancer cells to adapt to environmental challenges and cancer treatments. Mitochondria are extremely dynamic and controlled by mitochondrial biogenesis, mitophagy, fission, and fusion [6, 7]. Moreover, mitochondrial function and morphology are closely associated with cancer cell tumorigenicity, therapeutic resistance, and apoptosis susceptibility [8, 9].

Mitochondrial functions rely heavily on double-membrane structures. The outer mitochondrial membrane (OMM) is involved in apoptotic signaling, mitochondrial fusion and fission, and mitophagy. Permeabilization of the OMM, driven by recruitment of the pro-apoptotic proteins Bax and Bak, triggers the apoptotic cascade and leads to the activation of caspase-3, a key executor of apoptosis. The anti-apoptotic proteins Bcl-2 and Bcl-xL bind to and inhibit their pro-apoptotic activity. In contrast, the inner mitochondrial membrane (IMM) is characterized by low permeability and the presence of cristae, both of which are essential for energy generation. The mitochondrial contact site and cristae organizing system (MICOS) complex, comprising subunits such as MIC60 and MIC19, mediates IMM–OMM contact and plays a critical role in cristae formation and maintenance. Notably, opening of the IMM and OMM via the mitochondrial permeability transition pore (mPTP) disrupts the mitochondrial membrane potential and elicits diverse cellular responses from mitophagy to apoptosis [10–13]. However, the mechanisms by which glutamine metabolic stress regulates mitochondrial dynamics and the associated molecular targets remain largely unknown. In this study, we compared cell populations with distinct sensitivities to apoptosis induced by glutamine metabolism inhibition, described a novel function of adenine nucleotide translocase (ANT) in maintaining mitochondrial morphology, and developed a new avenue for cancer treatment.

## Results

### SLC25A6 mediates glutamine metabolic stress–induced apoptosis in colorectal cancer (CRC)

**Fig. 1:**
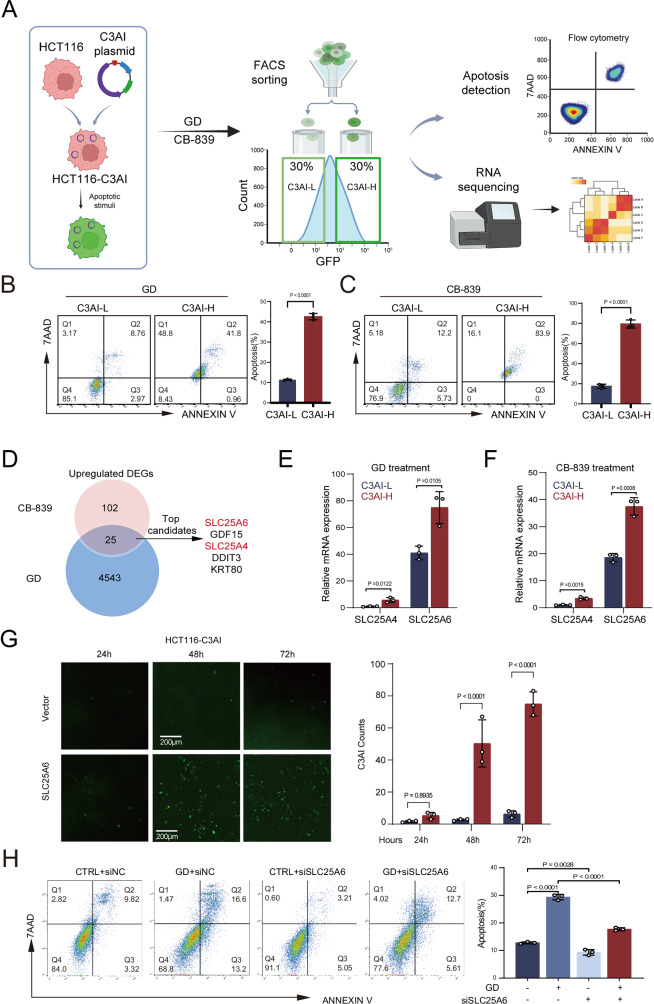
Identification of SLC25A6 as a mediator of glutamine metabolism inhibition-induced apoptosis. **A** Schematic of sorting strategy for HCT116-C3AI cells following GD. Cells were separated into C3AI-high (top 30% GFP-positive) and C3AI-low (bottom 30%) populations based on fluorescence intensity. **B**, **C** Flow cytometry analysis of apoptotic cells in C3AI-high and C3AI-low populations. **D** Overlap analysis of differentially expressed genes whose expression was upregulated between groups. **E**, **F** qPCR validation of SLC25A6 and SLC25A4 expression in C3AI-high and C3AI-low populations. **G** Caspase-3 activation in HCT116-C3AI cells overexpressing SLC25A6 compared with the vector control (scale bar: 200 μm). **H** Apoptosis analysis in control and SLC25A6-knockdown (KD) HCT116 cells under GD conditions. Data are presented as the mean ± SD (*n* = 3). Error bars represent the SD. Exact *P*-values are indicated.

To investigate the response of CRC cells to glutamine metabolic stress, we first screened different CRC cell lines using glutamine deprivation (GD) or the glutaminase inhibitor CB-839 and selected the HCT116 cell line for subsequent studies owing to its intermediate sensitivity (Supplementary Fig. 1A, B). To monitor apoptotic progression in individual cells, we stably transfected HCT116 cells with caspase-3 activity indicator (C3AI) [14], which rapidly exhibits fluorescence upon cleavage by activated caspase-3. We sorted cells challenged with glutamine metabolism inhibition based on C3AI fluorescence intensity: C3AI-high (top 30% GFP-positive) and C3AI-low (bottom 30%) (Fig. 1A). Consistent with C3AI function, C3AI-high cells showed a markedly higher apoptotic rate than C3AI-low cells, with an approximate four-fold increase (Fig. 1B, C). To minimize heterogeneity, we performed RNA sequencing on sorted populations. We identified 25 differentially expressed genes whose expressions were consistently upregulated across different treatments using stringent thresholds (fold change > 1.5, *P* < 0.05). The five most highly expressed genes were SLC25A6, GDF15, SLC25A4, DDIT3, and KRT80 (Fig. 1D). DDIT3 is a well-characterized regulator of GD-induced cell death [15, 16], confirming the validity of our experimental approach. Two members (SLC25A6 and SLC25A4) of the four-member ANT family were among the top five genes. Quantitative PCR (qPCR) validated the upregulation of SLC25A6 expression in C3AI-high cells (Fig. 1E, F). Considering the substantially higher basal levels in CRC cells and clinical specimens (Fig. 1E, F and Supplementary Fig. 1C), SLC25A6 cells were selected for further investigation. SLC25A6 overexpression significantly enhanced caspase-3 activation over time (Fig. 1G), whereas its knockdown (KD) markedly rescued GD-induced apoptosis in HCT116 cells (Fig. 1H). Collectively, these results indicate that SLC25A6 acts as a critical mediator of glutamine metabolism and stress-induced apoptosis in HCT116 cells.

### SLC25A6 suppresses CRC progression via apoptosis in vitro and in vivo

**Fig. 2:**
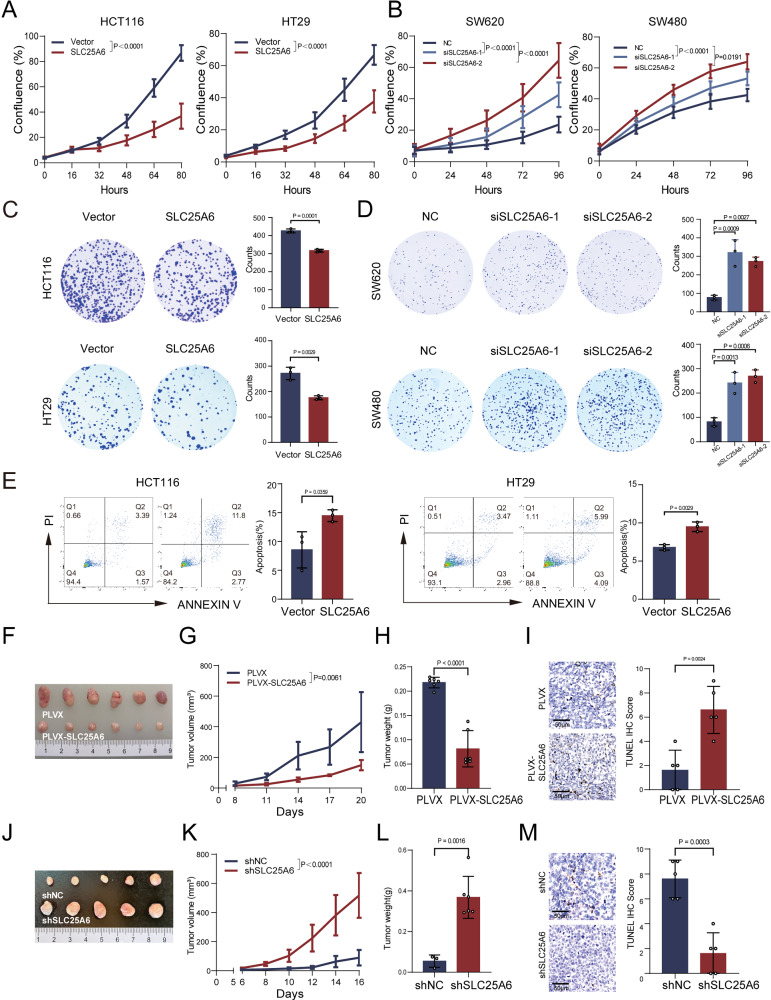
Antitumor function of SLC25A6 in vitro and in vivo. **A**, **B** Cell growth curves of control, SLC25A6-OE, and SLC25A6-KD cells measured using IncuCyte live-cell imaging. **C**, **D** Colony formation assays of control, SLC25A6-overexpression (OE), and SLC25A6-KD cells. **E** Flow cytometric analysis of apoptosis in control and SLC25A6-OE cells. Xenograft tumors from SLC25A6-OE or vector control cells: representative images (**F**) tumor volume growth curves (**G**) and weights (**H**) (*n* = 5 mice per group). **I** TUNEL IHC staining of xenografts (scale bar: 50 μm). Xenograft tumors with SLC25A6 KD: representative images (**J**), tumor volume growth curves (**K**) and weights (**L**) (*n* = 5 mice per group). **M** TUNEL IHC staining of KD xenografts (scale bar: 50 μm). Data are presented as the mean ± SD (*n* = 3). Error bars represent the SD. Exact *P*-values are shown.

To characterize the role of SLC25A6 in CRC, we first assessed its expression in a panel of CRC cell lines by qPCR and western blotting (Supplementary Fig. 2A). Transient overexpression (OE) and KD models were established in different CRC cell lines (Supplementary Fig. 2B). Functional assays showed that SLC25A6 OE significantly inhibited cell growth, whereas SLC25A6 KD markedly promoted cell growth, as demonstrated by confluence-based growth assessment (Fig. 2A, B) and colony formation assays (Fig. 2C, D). Flow cytometry revealed that SLC25A6 OE significantly enhanced apoptosis (Fig. 2E), which was reversed by the pan-caspase inhibitor Z-VAD in a dose-dependent manner (Supplementary Fig. 2C).

To validate these in vitro findings in vivo, we investigated SLC25A6 function using a xenograft nude mouse model established with transfected cell lines showing stable SLC25A6 OE or KD (Supplementary Fig. 2E). Forced expression of SLC25A6 significantly impaired tumor growth, as evidenced by reduced tumor volume and weight (Fig. 2F–H). Conversely, stable SLC25A6 KD via shRNA transfection accelerated tumor progression and increased the tumor mass (Fig. 2J–L). Immunohistochemistry (IHC) revealed an increase in the number of TUNEL-positive tumor cells in xenografts derived from SLC25A6 OE cells (Fig. 2I), whereas the number of TUNEL-positive cells was decreased in xenografts derived from SLC25A6 KD cells (Fig. 2M). These results demonstrate that SLC25A6 functions as a tumor suppressor in CRC by promoting apoptosis both in vitro and in vivo.

### SLC25A6 induces mitochondrial dysfunction without increasing mPTP opening

**Fig. 3:**
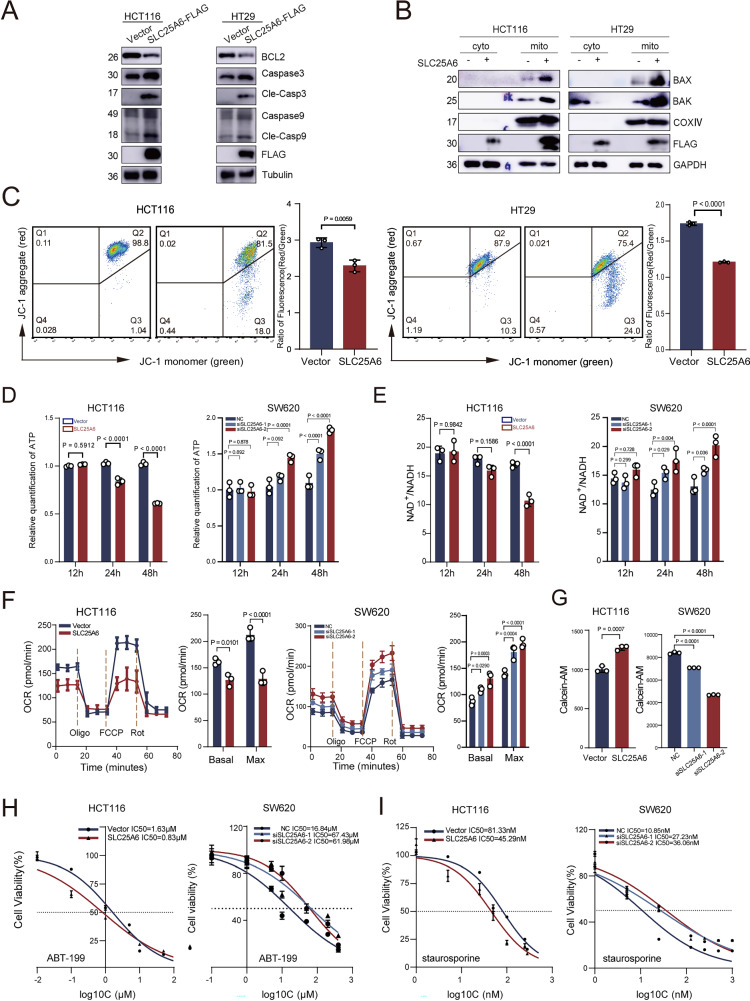
Mitochondrial dysfunction in SLC25A6-OE cells. **A** Representative images of immunoblotting for cleaved caspase-9, cleaved caspase-3, and Bcl-2 in control and SLC25A6-OE cells. **B** Representative images of Bax and Bak immunoblotting in mitochondrial and cytoplasmic fractions of control and SLC25A6-OE cells. **C** JC-1 staining of mitochondrial membrane potential in control and SLC25A6-OE cells. **D** Quantification of cellular ATP levels. **E** Quantification of NAD⁺/NADH ratio. **F** Oxygen consumption rate measured using Seahorse assay. **G** Quantification of mPTP opening. Cell viability assays following treatment with ABT-199 (**H**) or staurosporine (**I**). Data are presented as the mean ± SD (*n* = 3). Error bars represent the SD. Exact *P*-values are shown.

To elucidate the pro-apoptotic mechanism of SLC25A6, we examined the activation of caspases and Bcl-2 family members—key regulators of the intrinsic apoptotic pathway. SLC25A6 OE increased the cleaved forms of caspase-9 and caspase-3 (Fig. 3A), elevated the levels of Bax and Bak in mitochondrial extracts (Fig. 3B), and reduced the expression of Bcl-2 (Fig. 3A), collectively indicating activation of the intrinsic apoptotic pathway. In addition, SLC25A6-OE cells exhibited pronounced mitochondrial dysfunction, characterized by loss of mitochondrial membrane potential (JC-1 assay), reduced ATP generation, decreased NAD⁺/NADH ratio, and impaired oxidative phosphorylation (measured as oxygen consumption rate). Conversely, SLC25A6 knockdown led to the opposite effects (Fig. 3C–F and Supplementary Fig. 3A–C). To verify whether above changes in pro-apoptosis and mitochondrial dysfunction are due to the increase of mitochondrial permeability, we analyzed mPTP opening using a quenching method. However, it revealed a slight increase in fluorescence, indicating a modest decrease rather than an increase in mPTP opening in SLC25A6-OE cells (Fig. 3G and Supplementary Fig. 3D). Notably, SLC25A6 OE sensitized CRC cells to mitochondria-targeting apoptotic stimuli, including the Bcl-2 inhibitor ABT-199 and staurosporine (Fig. 3H, I and Supplementary Fig. 3E, F), whereas no significant effects were observed with conventional chemotherapeutic agents, such as 5-fluorouracil or oxaliplatin (Supplementary Fig. 3G, H). These findings demonstrate that SLC25A6 promotes intrinsic apoptosis and induces mitochondrial dysfunction without increasing mPTP opening.

### SLC25A6 mediates GD-related mitochondrial fragmentation via mitofission

**Fig. 4:**
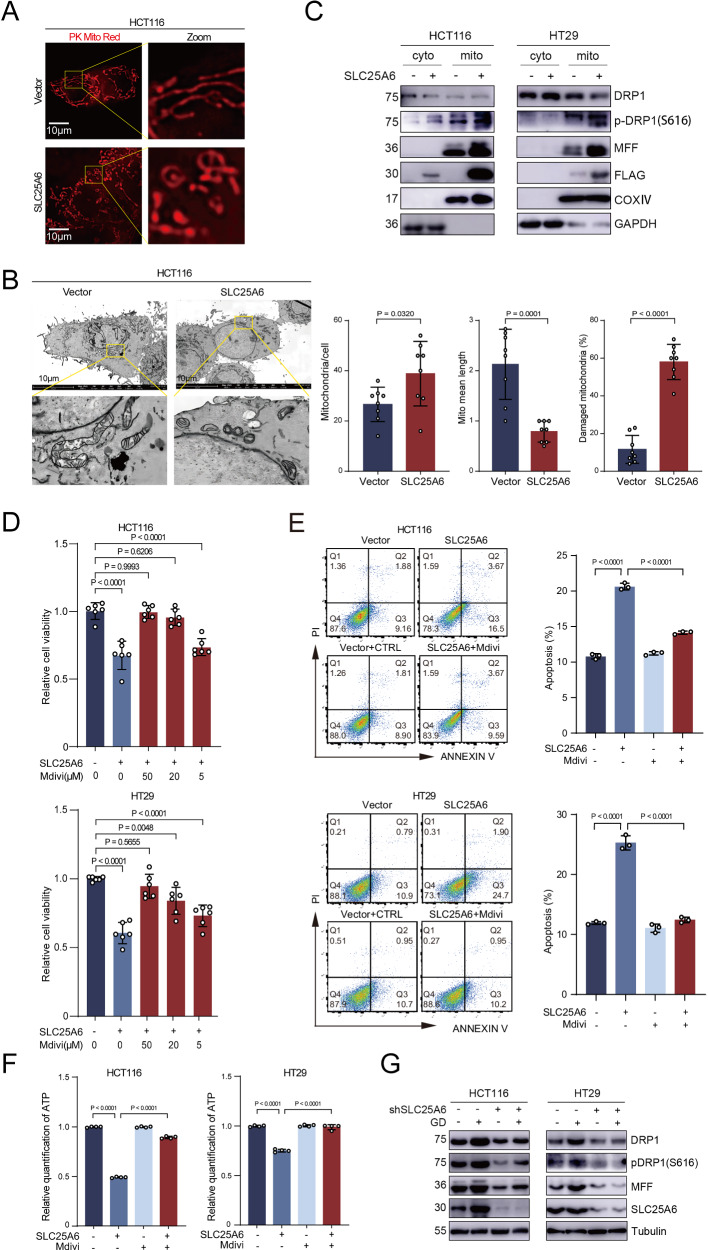
SLC25A6 promotes mitochondrial fission. **A** Representative immunofluorescence staining images of mitochondrial morphology in control and SLC25A6-OE cells (scale bar: 10 μm). **B** Representative TEM images of mitochondrial ultrastructure in HCT116 control and SLC25A6-OE cells (scale bar: 10 μm). **C** Representative images of immunoblots for mitochondrial fission proteins DRP1 and MFF in control and SLC25A6-OE cells. **D** Viability of SLC25A6-OE cells treated with increasing concentrations of Mdivi. Quantification of apoptosis (**E**) and ATP levels (**F**) in SLC25A6-OE cells treated with vehicle or Mdivi-1. **G** Representative images of immunoblots for mitochondrial fission proteins in CRC cells cultured under GD with or without SLC25A6 KD. Data are presented as the mean ± SD (*n* = 3). Error bars represent the SD. Exact *P*-values are shown.

To elucidate the mechanism underlying SLC25A6-induced mitochondrial dysfunction, we first evaluated mitochondrial morphology. Immunofluorescence analysis with PK mito red fluorescent probe revealed that SLC25A6 overexpression transformed the mitochondrial morphology from interconnected tubular networks into fragmented punctate structures (Fig. 4A). Transmission electron microscopy (TEM) confirmed severe ultrastructural damage, including disrupted cristae and the emergence of characteristic “donut-shaped” mitochondria (Fig. 4B). Conversely, SLC25A6-KD cells exhibited enlarged mitochondria with abundant and intact cristae, consistent with enhanced mitochondrial integrity (Supplementary Fig. 4C). To verify whether the morphological changes of mitochondria result from mitochondrial dynamics, we examined the markers associated with mitochondrial fission, fusion and mitophagy. Western blot analysis demonstrated selective upregulation of the expression of mitochondrial fission proteins such as DRP1 and MFF (Fig. 4C), whereas no significant changes were observed in proteins related to mitochondrial fusion (MFN1/2) or biogenesis (PGC1α, TFAM) (Supplementary Fig. 4A). To explore mitophagy involvement, we measured PINK1/Parkin phosphorylation and performed TEM but detected no significant changes (Supplementary Fig. 4B and Fig. 3C). Pharmacological inhibition of mitofission with Mdivi-1 dose-dependently rescued SLC25A6-induced growth inhibition (Fig. 4D) and apoptosis (Fig. 4E) and reduced ATP levels (Fig. 4F), indicating that aberrant mitofission contributed to SLC25A6-related mitochondrial damage and apoptosis. Moreover, GD induced pronounced mitochondrial fragmentation and increased the expression of mitofission markers (Supplementary Fig. 4D, E), which was largely abrogated by SLC25A6 KD (Fig. 4G). These findings indicate that SLC25A6 induces mitofission and mediates GD-induced mitochondrial fragmentation.

### Disruption of MIC60–MIC19 interaction by SLC25A6 contributes to mitofission

**Fig. 5:**
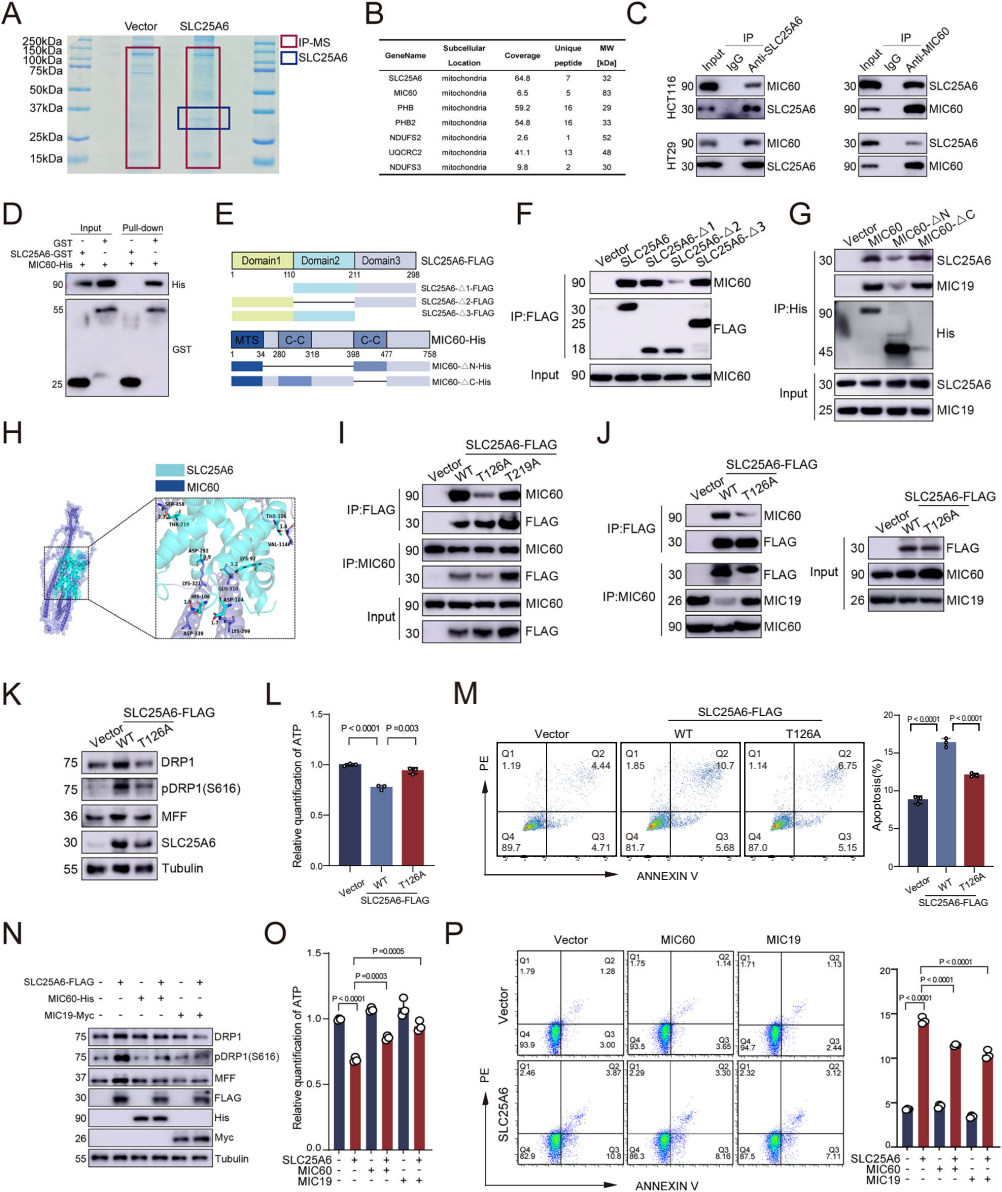
SLC25A6 binds to MIC60 and disrupts the MIC60–MIC19 complex. **A**, **B** Immunoprecipitation–mass spectrometry of SLC25A6 interactome showing enrichment of mitochondrial structural proteins and OXPHOS components. **C** Co-IP analysis confirming the interaction between SLC25A6 and MIC60. **D** GST pull-down and immunoblotting analysis to detect SLC25A6-GST and MIC60 binding. **E** Schematic representation of domain-truncated mutants of SLC25A6 and MIC60. **F**, **G** Co-IP of wild-type and truncated mutants showing interaction domains. **H** Molecular docking prediction of key residues mediating interaction between SLC25A6 and MIC60. **I** Co-IP of wild-type SLC25A6 and point mutant with MIC60. **J** Co-IP of wild-type and T126A mutant SLC25A6 at MIC60 and MIC19. **K** Representative images of immunoblots for mitochondrial fission proteins in wild-type and T126A mutant SLC25A6-OE cells. **L** Measurement of cellular ATP levels in wild-type and T126A mutant SLC25A6-OE cells (*n* = 3). **M** Flow cytometry of apoptosis in wild-type and T126A mutant SLC25A6-OE cells (*n* = 3). SLC25A6-overexpressing cells were further subjected to restoration of MIC60 or MIC19 expression to examine **N** mitochondrial fission-related markers, **O** cellular ATP levels, and **P** apoptosis. Data are presented as the mean ± SD. Error bars represent the SD. Exact *P*-values are shown.

To explore the mechanism of SLC25A6-induced mitofission, we performed immunoprecipitation–mass spectrometry analysis on SLC25A6-OE cells. Mitochondrial structural regulators (MIC60, PHB, and PHB2) and OXPHOS components (NDUFS2, NDUFS3, and UQCRC2) were the predominant interactors (Fig. 5A, B and Supplementary Fig. 5A). Among these interacting proteins, MIC60 drew our attention due to its core role in the MICOS complex essential for cristae organization [17–19]. We validated the direct physical interaction between SLC25A6 and MIC60 by co-immunoprecipitation and further confirmed their direct binding using an in vitro GST pull-down assay (Fig. 5C, D). To elucidate the structural basis of this interaction, we generated a series of domain-truncated SLC25A6 and MIC60 mutants (Fig. 5E). IP assay revealed that deletion of domain 2 (residues 110–211) in SLC25A6 (Δ2) or deletion of the N-terminus of MIC60 (ΔN) substantially abrogated their interaction (Fig. 5F, G). Molecular docking analysis identified two key residues (T126 and T219) within or adjacent to domain 2 of SLA25A6 at the binding interface (Fig. 5H and Supplementary Fig. 5B). The SLC25A6 T126A mutant, but not the T219A mutant, failed to bind to MIC60 (Fig. 5G).

Given that the MIC60–MIC19 association is indispensable for MICOS complex integrity [20, 21], we hypothesized that SLC25A6 competes with MIC19 to bind with MIC60. Similarly, MIC60 ΔN significantly inhibited MIC60–MIC19 binding, suggesting that MIC19, similar to SLC25A6, binds to the N-terminus of MIC60 (Fig. 5G). Furthermore, SLC25A6 OE markedly reduced MIC60–MIC19 binding (Fig. 5J). Notably, compared to wild-type SLC25A6, the T126A mutant failed to reduce the MIC60–MIC19 association and consequently ceased to promote mitofission, reduce intracellular ATP production, and induce apoptosis (Fig. 5K–M). These findings demonstrate that residue T126 of SLC25A6 is critical for its interaction with MIC60; disruption of the MIC60–MIC19 association by SLC25A6 contributes to mitochondrial dysfunction and, ultimately, apoptosis.

### Low expression of SLC25A6 is associated with poor prognosis in CRC

**Fig. 6:**
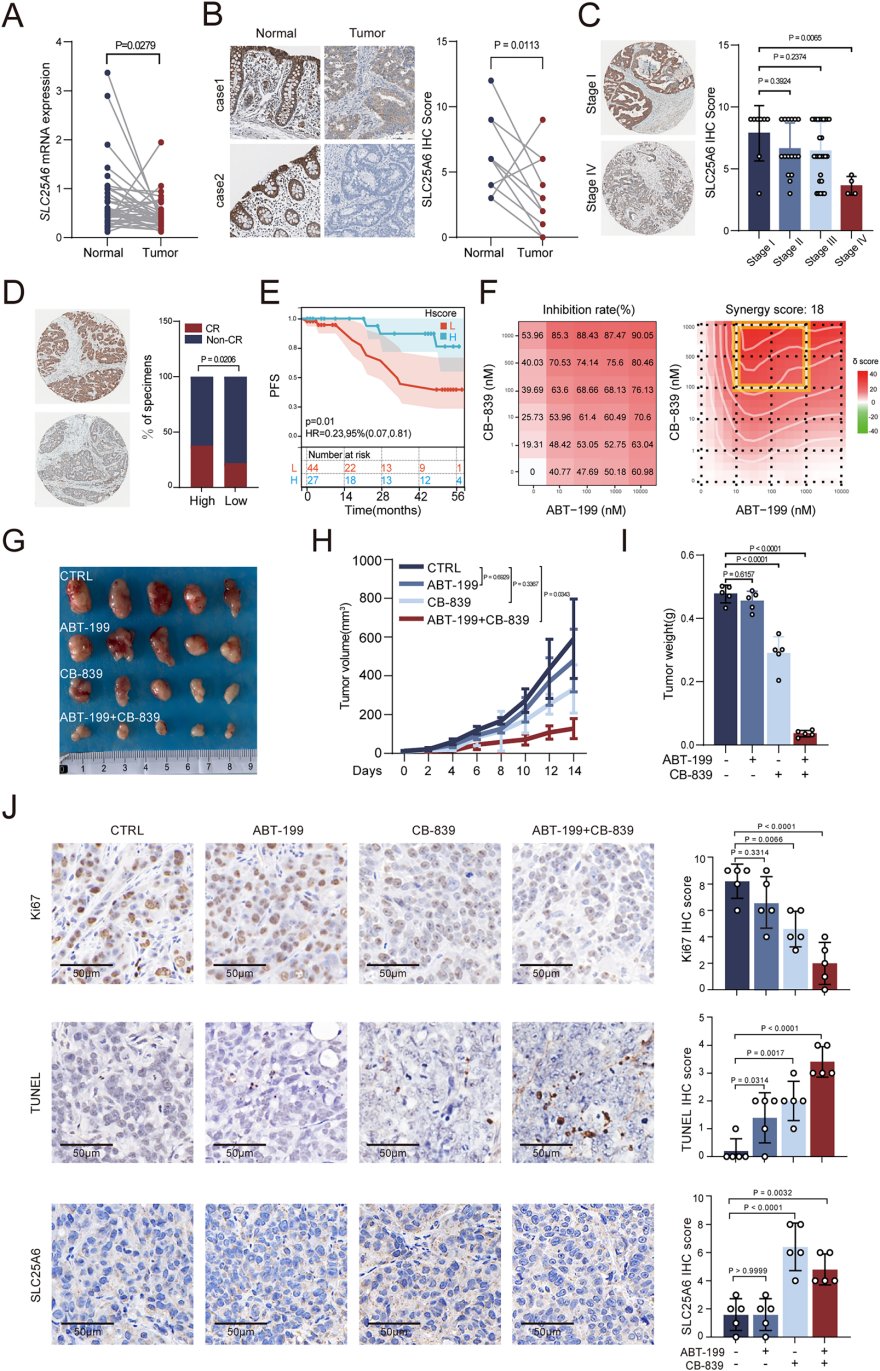
SLC25A6 enhancement by glutaminase inhibitor synergizes with the antitumor effect of Bcl-2 inhibition. **A**, **B** Comparison between paired CRC and adjacent normal tissues for SLC25A6 mRNA (A, *n* = 37 pairs) and protein levels (B, *n* = 12 pairs). **C** Comparisons between different clinical stages of CRC for SLC25A6 expression (stage I, *n* = 8; stage II, *n* = 16; stage III, *n* = 28; stage IV, *n* = 4). **D** Distribution of treatment responses in a clinical cohort of colorectal cancer patients receiving chemoradiotherapy, stratified by SLC25A6 expression levels. **E** Kaplan–Meier survival analysis of patients with CRC stratified by SLC25A6 expression. **F** Analysis of CB-839 and ABT-199 synergy in HT29 cells (*n* = 3). Representative xenograft images (**G**) tumor growth curves (**H**) and tumor weights (**I**) in mice treated with vehicle, CB-839, ABT-199, or combination treatment (*n* = 5 mice/group). **J** Immunohistochemical staining of Ki67, TUNEL, and SLC25A6 in xenografts (*n* = 5; scale bar: 50 μm). Data are presented as the mean ± SD. Error bars represent the SD. Exact *P*-values are shown.

To test the clinical relevance, we examined SLC25A6 expression in CRC specimens. SLC25A6 mRNA and protein levels were markedly downregulated in 70% (26/37) and 75% (9/12) of tumor tissues, respectively, compared to those in paired adjacent normal mucosa (Fig. 6A, B and Supplementary Table 1, Cohort 1). SLC25A6 expression progressively decreased with advancing clinical stage (stage I, *n* = 8; stage II, *n* = 16; stage III, *n* = 28; stage IV, *n* = 4; Fig. 6C and Supplementary Table 1, Cohort2), was significantly lower in poorly differentiated tumors than in moderately or well-differentiated tumors (Supplementary Fig. 6A), and was further reduced in liver metastases compared with matched primary CRC tumors (Supplementary Fig. 6B) (*n* = 12, Supplementary Table 1, Cohort4). Furthermore, among CRC patients receiving chemoradiotherapy, individuals with high SLC25A6 expression exhibited a significantly increased proportion of complete response compared with those with low SLC25A6 expression (Fig. 6D) (*n* = 53, Supplementary Table 1, Cohort 5). In a cohort of 163 patients with CRC (Supplementary Table 1, Cohort 3), low SLC25A6 protein expression was significantly correlated with an unfavorable prognosis (Fig. 6E), highlighting its potential as a prognostic biomarker. These findings demonstrate that downregulation of SLC25A6 expression is associated with CRC progression and poor prognosis.

### Synergistic antitumor effect of GD and Bcl-2 inhibition in CRC

Because the inhibition of glutamine metabolism upregulates SLC25A6 expression (Fig. 1), we hypothesized that the glutaminase inhibitor CB-839 would induce mitochondrial dysfunction and exert a synergistic effect with mitochondria-targeting agents such as the Bcl-2 inhibitor ABT-199. We found that CB-839 treatment promoted mitochondrial fission, activated fission-related protein markers and reduced ATP levels (Supplementary Fig. 6C–E). In vitro, this dual treatment exhibited strong synergistic antitumor effects in various CRC cell lines (HT29, synergy score: 18; HCT116, synergy score: 12.48; Fig. 6F and Supplementary Fig. 6F). In the subcutaneous xenograft model (Supplementary Fig. 6G), combined treatment markedly suppressed tumor growth and reduced tumor weight compared to that seen with individual agents (Fig. 6G–I). Immunohistochemical staining revealed that CB-839 significantly increased SLC25A6 expression, whereas the combination treatment was associated with the lowest Ki67 levels and highest TUNEL positivity (Fig. 6J). Furthermore, no detectable weight loss or histopathological damage to major organs in mice was observed under the combination strategy (Supplementary Fig. 6H, I), supporting its safety and therapeutic potential. These findings indicate that dual GD and Bcl-2 inhibition is a promising strategy for suppressing CRC growth by enhancing apoptosis.

## Discussion

The plasticity and heterogeneity of cancer cells severely limit the clinical efficacy of glutamine metabolism inhibitor (GMI) therapies. In this study, single-cell apoptosis monitoring revealed distinct cellular responses to GD stress and validated the pivotal role of SLC25A6 in mitochondria-associated cell death by regulating mitochondrial dynamics. SLC25A6 belongs to the ANT family, which includes nuclear-encoded proteins localized to the IMM. In humans, this family comprises four members encoded by SLC25A4 (ANT1), SLC25A5 (ANT2), SLC25A6 (ANT3), and SLC25A31 (ANT4) [22]. Previous studies suggested isoform-specific functions for ANT proteins, with SLC25A6 exhibiting pro-apoptotic potential in a cell type- and stimulus-dependent manner [23]. However, the mechanism by which ANT family members regulate cell death remains largely unknown.

ANTs are central to bioenergetic metabolism, responsible for ADP/ATP exchange across the IMM [24], and have been implicated in mitophagy [25] and potentially mPTP opening [13]. In our study, SLC25A6 expression was significantly upregulated in GMI-sensitive CRC cells. However, its pro-apoptotic effect was independent of its well-characterized functions. Treatment with the ADP/ATP exchange inhibitors bongkrekic acid [24] and carboxyatractyloside [26] had no impact on SLC25A6 overexpression-induced apoptosis (data not shown). Additionally, we observed no effect of SLC25A6 on mitophagy in the experimental system. Although ANTs participate in mPTP regulation and may influence its composition [27], we found no evidence of increased mPTP opening following SLC25A6 overexpression, further indicating that SLC25A6 exerts its pro-apoptotic role via a non-canonical pathway.

Notably, we uncovered a novel function of SLC25A6: disruption of the MICOS complex. The formation and maintenance of mitochondrial cristae are highly dependent on an intact MICOS assembly [21]. The depletion of either MIC60 or MIC19 alone is sufficient to trigger MICOS disassembly and severe mitochondrial fragmentation [19]. We demonstrated that SLC25A6 binds directly to MIC60, disrupting MIC60–MIC19 interactions and promoting MICOS complex dissociation. Critically, the SLC25A6 T126A mutant, which lacks the ability to interact with MIC60, failed to induce mitochondrial fragmentation and apoptosis. These results define a previously unrecognized role of SLC25A6 in the regulation of mitochondrial ultrastructure and morphology. Furthermore, protein sequence alignment using NCBI BLAST revealed that the T126 residue is highly conserved across ANT family members (data not shown). Given that SLC25A6 is abundantly expressed in the colon, we focused on its role in GD-induced apoptosis in CRC. However, whether other ANT isoforms have similar functions in other tissues requires further investigation.

Mitochondrial dynamics respond rapidly to metabolic stress. Accumulating evidence indicates that the activities of fusion and fission machineries are regulated by metabolic signals primarily at the post-translational level [28]. Acute glutamine starvation induces mitochondrial fusion within hours [29], whereas persistent glutamine deficiency results in mitochondrial fragmentation [30]. Here, we identified a novel mechanism: glutamine metabolic stress induces mitofission via the upregulation of the expression of the IMM-located SLC25A6 protein. This mechanism also revealed a new vulnerability of tumor cells following GMI treatment—increased sensitivity to intrinsic apoptosis. Similarly, GMI synergized with the Bcl-2 blockade (via ABT-199), resulting in enhanced apoptosis and tumor suppression in vivo. These findings suggest a clinically actionable strategy for combining metabolic stress inducers with apoptosis-targeting agents to improve therapeutic efficacy. However, the absence of unbiased techniques—such as single-cell sequencing—restricted our ability to unravel the molecular mechanisms underlying the heterogeneous responses to metabolic stress.

It should be noted that the current study only delineated the mechanism by which SCL25A6 regulates mitochondrial morphology, function, and apoptosis sensitivity based on cell line experiments. Whether this mechanism is functionally relevant under physiological and pathological conditions in vivo remains to be further validated by more in-depth studies. In addition, the relationship between the canonical and novel functions of SLC25A6 warrants further exploration. The upstream regulatory signals controlling SCL25A6 expression also remain to be elucidated. Furthermore, the detailed molecular mechanism by which glutamine starvation modulates SLC25A6 expression, an initial observation in this study, has not been fully dissected and requires further investigation.

In conclusion, we identified SLC25A6 as a tumor suppressor that links glutamine metabolism to mitochondrial apoptosis via MIC60 interaction and excessive mitochondrial fission. These findings not only advance our understanding of metabolic stress responses in CRC but also provide a preclinical rationale for combination therapies targeting both cellular metabolism and apoptosis pathways.

## Materials and methods

### Reagents

CB-839 (HY-12248), ABT-199 (HY-15531), Oxaliplatin (HY-17371), 5-FU (HY-90006), Z-VAD-FMK (HY-16658B), and Mdivi (HY-15886) were purchased from MedChemExpress (Shanghai, China). The Cell Counting Kit-8 (CK-04) and PK Mito Red (PKMR-1) were purchased from Dojindo (Kumamoto, Japan) and GenVivo (Shanghai, China), respectively.

### Tissue specimens and cell lines

CRC and adjacent non-tumor tissues were obtained from surgical FFPE specimens. All patients were admitted to our center between 2018 and 2019. No patients received neoadjuvant therapy or exhibited other malignant tumors. This study was approved by the Ethics Committee of the Cancer Institute (Hospital), Chinese Academy of Medical Sciences (CAMS) & Peking Union Medical College (PUMC) (approval no. 21/105-2776), and performed in accordance with the Declaration of Helsinki. Written informed consent was obtained from all patients before sample collection. Detailed patient information is provided in Supplementary Table 1.

Human CRC cell lines (RKO, DLD1, HCT116, HT29, SW480, and SW620) were obtained from the American Type Culture Collection (Manassas, VA, USA). Cells were maintained in DMEM (HyClone, Logan, UT, USA) supplemented with 10% fetal bovine serum (Cell Technologies, Beijing, China) and 1% penicillin/streptomycin (Gibco, Carlsbad, CA, USA) in a humidified incubator at 37 °C with 5% CO₂.

### Transcriptome sequencing and data analysis

Total RNA was isolated from HCT116 cells under GD or CB-839 treatment using TRIzol reagent (Invitrogen, Carlsbad, CA, USA). RNA quality and integrity were evaluated using the Agilent 2100 Bioanalyzer (Agilent Technologies, Santa Clara, CA, USA), and samples with RNA integrity number (RIN) ≥ 7.0 were used for subsequent analyses. Sequencing libraries were constructed using a Hieff NGS Ultima Dual-mode mRNA Library Prep Kit (13335ES, Yeasen Biotechnology, Shanghai, China) and sequenced on an Illumina platform. Bioinformatic analysis of raw reads was performed using the BMKCloud platform (www.biocloud.net).

### Bioinformatics analysis

We analyzed the levels of ANT family members (SLC25A4, SLC25A5, SLC25A6, and SLC25A31) and compared their expression profiles using RNA sequencing data for colorectal adenocarcinoma and rectal adenocarcinoma downloaded from The Cancer Genome Atlas database.

### siRNA transfection

siRNAs targeting SLC25A6 were designed and synthesized by SinoGenoMax (Beijing, China). Non-targeting scrambled siRNA with no homology to any known human gene sequence was used as a negative control (siNC, SinoGenoMax). Transfection was conducted using JetPRIME reagent (PolyPlus, Illkirch, France) according to the manufacturer’s instructions. RNA and proteins were extracted 48 h post-transfection. The siRNA sequences are listed in Supplementary Table 2.

### Plasmids and establishment of stable cell lines

Expression plasmids (pcDNA3.1-3×FLAG, pcDNA3.1-SLC25A6-3×FLAG, deletion and mutant constructs, MIC19 and MIC60 plasmids) and lentiviral vectors (pLVX-IRES-Neo-3×FLAG, pLVX-IRES-Neo-3×FLAG-SLC25A6, PLKO.1-EGFP-PURO-shNC, and PLKO.1-EGFP-PURO-shSLC25A6) were synthesized by Mailgene Biosciences (Beijing, China) and confirmed by sequencing. The plasmid encoding C3AI [14] was kindly provided by Prof. Binghui Li. Lentiviruses were produced by co-transfecting HEK-293FT cells with the transfer vectors and packaging plasmids pMD2.G and psPAX.2. CRC cells were infected with the lentivirus using HiTransG P reagent (GeneChem, Suzhou, China) and selected using puromycin to establish stable cell lines.

### Reverse transcription PCR and qPCR

Total RNA was extracted from CRC cells and tissues using an RNA purification kit (Vazyme Biotech, Nanjing, China). cDNA was synthesized using HiScript III All-in-One RT SuperMix (Vazyme). The cDNA products were subjected to 40 cycles of PCR. qPCR was performed using TB Green Premix Ex Taq (RR420A; Takara Bio, Kusatsu, Japan) on an ABI QuantStudio DX system (Applied Biosystems, Foster City, CA, USA). Relative mRNA expression was calculated using the 2^–ΔΔCt^ method with 18S rRNA serving as the internal control. The primer sequences are listed in Supplementary Table 3.

### IC₅₀ assay

CRC cells (5 × 10³ per well) were seeded in 96-well plates, and proliferation was monitored in terms of confluence (%) every 12 h using an IncuCyte Live-Cell Analysis System (Sartorius, Göttingen, Germany). IC₅₀ values were calculated from dose–response curves fitted with nonlinear regression (log[inhibitor] vs. response, variable slope) using GraphPad Software, San Diego, CA, USA.

### Cell viability assay

CRC cells (5 × 10³ per well) were seeded in 96-well plates, and proliferation was monitored in terms of confluence (%) every 12 h using the IncuCyte Live-Cell Analysis System (Sartorius).

### IHC

Paraffin-embedded slides were incubated with primary antibodies at 4 °C overnight, followed by a Mouse/Rabbit Enhanced Polymer System (PV-9000, ZSGB-BIO, Beijing, China) and visualized with DAB (ZLI-9017, ZSGB-BIO). Tissue microarrays were scanned using a NanoZoomer digital scanner (Hamamatsu Photonics, Hamamatsu, Japan). Immunoreactive scores were calculated as the intensity(0–3) × proportion of positive cells(0–3). The IHC antibodies are listed in Supplementary Table 4.

### Mitochondrial and cytoplasmic fractionation

Mitochondrial and cytoplasmic fractions were separated from cell pellets using a mitochondrial isolation kit (Beyotime, Shanghai, China) according to the manufacturer’s instructions.

### ATP assay

Cellular ATP levels were measured with a CellTiter-Glo Luminescent Assay (Promega, Madison, WI, USA). Following treatment, CellTiter-Glo reagent was added to each well, and luminescence was detected using a microplate reader.

### Measurement of mitochondrial membrane potential

Mitochondrial membrane potential was assessed using JC-1 staining (Beyotime). Cells (1 × 10⁶) were incubated with JC-1 at 37 °C for 15 min and analyzed by flow cytometry. Red and green fluorescence were detected with PE and FITC channels, respectively.

### Assessment of mPTP opening

mPTP opening was evaluated with an mPTP Assay Kit (Beyotime) based on the Calcein-AM/cobalt ion (Co²⁺) quenching method. Cells were incubated with Calcein-AM and a cobalt quencher, and the residual fluorescence (inversely correlated with mPTP opening) was quantified using flow cytometry.

### Apoptosis assay

Apoptosis was detected using Annexin V FITC/PI (AD10, Dojindo) or Annexin V/7-AAD (4 A Biotech, Shanghai, China) apoptosis detection kits, according to the manufacturer’s instructions, followed by flow cytometry.

### TEM

Cells were fixed with 3% glutaraldehyde at 4 °C for 2 h or overnight, dehydrated with graded ethanol and propylene oxide, and embedded in epoxy resin. Ultrathin sections were prepared and examined using a TEM-1400 Plus electron microscope (Talos L120c, Thermo Fisher Scientific, Waltham, MA, USA).

### Western blotting

Proteins were extracted with RIPA buffer (C1053, Applygen Technologies, Beijing, China) containing protease (B14001, Bimake, Shanghai, China) and phosphatase inhibitors (B15001, Bimake). Concentrations were determined using a BCA Protein Assay Kit (23225, Thermo Fisher Scientific). The antibodies (working dilutions) are listed in Supplementary Table 4.

### Immunoprecipitation and mass spectrometry

For exogenous co-immunoprecipitation, HCT116 or HT29 cells were transfected with FLAG-tagged plasmids. Lysates were incubated with anti-FLAG magnetic beads (MedChemExpress) overnight at 4 °C. The complexes were eluted by boiling, analyzed using western blotting, or separated via SDS-PAGE and submitted for mass spectrometry (BIOMS, Shanghai, China). Endogenous co-immunoprecipitation was performed by incubating cell lysates with specific antibodies and Protein A/G magnetic beads (HY-K0202, MedChemExpress) for 2 h, followed by overnight rotation at 4 °C. The complexes were eluted and analyzed by western blotting.

### GST pull-down

GST pull-down assays were performed to examine the direct interaction between SLC25A6 and MIC60 in vitro. The full-length human SLC25A6 coding sequence was cloned into the pGEX-4T-1 vector to generate a GST-tagged fusion protein, while full-length human MIC60 was cloned into the pET-28a vector to express a His-tagged protein. All constructs were verified by DNA sequencing. Recombinant proteins were expressed in *Escherichia coli* BL21 (DE3) cells. After induction, bacterial cells were harvested and lysed, and recombinant proteins were purified using a commercial kit according to the manufacturer’s instructions (Beyotime). For pull-down assays, purified GST-SLC25A6 protein was incubated with purified His-MIC60 protein at 4 °C overnight with gentle rotation. Subsequently, GST magnetic beads were added and incubated at 4 °C for an additional 2 h to capture GST-tagged proteins and associated binding proteins.

### Molecular docking

The 3D structures of MIC60 and ADT3 (SLC25A6) were obtained from the AlphaFold Protein Structure Database (https://alphafold.ebi.ac.uk/). Protein–protein docking was performed using the HDOCK server, and the resulting complexes were visualized using PyMOL (v2.6, Schrödinger, LLC, New York, NY, USA).

### Seahorse metabolic assay

HCT116 or HT29 cells (1 × 10⁴ per well) were seeded in XF96 plates (Seahorse Bioscience, North Billerica, MA, USA) and cultured overnight. Oxygen consumption rate was measured with a Seahorse XFe-96 Analyzer (Agilent Technologies, Santa Clara, CA, USA) after sequential injections of oligomycin (1.5 μM), FCCP (0.5 μM), and rotenone/antimycin A (0.5 μM each). Data were analyzed using WAVE software (Agilent Technologies) and normalized to cell numbers.

### Fluorescence microscopy

Cells were grown on confocal dishes, washed with PBS, and stained with PK Mito, according to the manufacturer’s protocol. Images were acquired with a super-resolution microscope (DeltaVision OMX SR, GE Healthcare, Chicago, IL, USA).

### Tumor xenograft model

Male BALB/c nude mice (6 weeks old) were purchased from Huafukang Biotechnology Co., Ltd. (Beijing, China) and maintained under SPF conditions. After acclimatization, 2 × 10⁶ CRC cells were injected subcutaneously into the right flank. Mice were randomized into four groups (*n* = 5 per group): vehicle control; ABT-199 (50 mg/kg/d, intraperitoneal); CB-839 (200 mg/kg/d, oral gavage); and combination treatment. Tumor volume was measured every 3 d and calculated as (length × width²)/2. After 12 d, the mice were euthanized by CO₂ asphyxiation followed by cervical dislocation to ensure death; thereafter, tumors were excised, weighed, and analyzed using IHC. All animal studies were approved by the Animal Center of the National Cancer Center/Cancer Hospital, CAMS & PUMC (approval no. NCC2021A264).

### Statistical analysis

All experiments were independently repeated at least thrice. Statistical analyses were performed using GraphPad Prism (v9.0). Data distribution normality was assessed using the Shapiro–Wilk test, and homogeneity of variance was evaluated using Levene’s test. Comparisons between two groups were conducted using unpaired Student’s *t*-tests, whereas multiple group comparisons were performed using one-way or two-way analysis of variance followed by Tukey’s post-hoc test. Data are presented as the mean ± SD. Statistical significance was set at *P* < 0.05.

## Supplementary information

supplementary data

original data

Supplementary Table 1

Supplementary Table 2

Supplementary Table 3

Supplementary Table 4

## Data Availability

The data, analytic methods, and study materials will be made available to other researchers as required.
