# Supplementary material for: Glutamine metabolic stress induces SLC25A6-dependent mitofission via MIC60–MIC19 complex disassembly in colorectal cancer
Source: Cell Death Dis. 2026 Apr 23;17(1):537. doi: 10.1038/s41419-026-08754-6 (PMC13237379; doi:10.1038/s41419-026-08754-6)
Supplement: Supplementary file 1 — supplementary data [file 41419_2026_8754_MOESM1_ESM.docx]

**
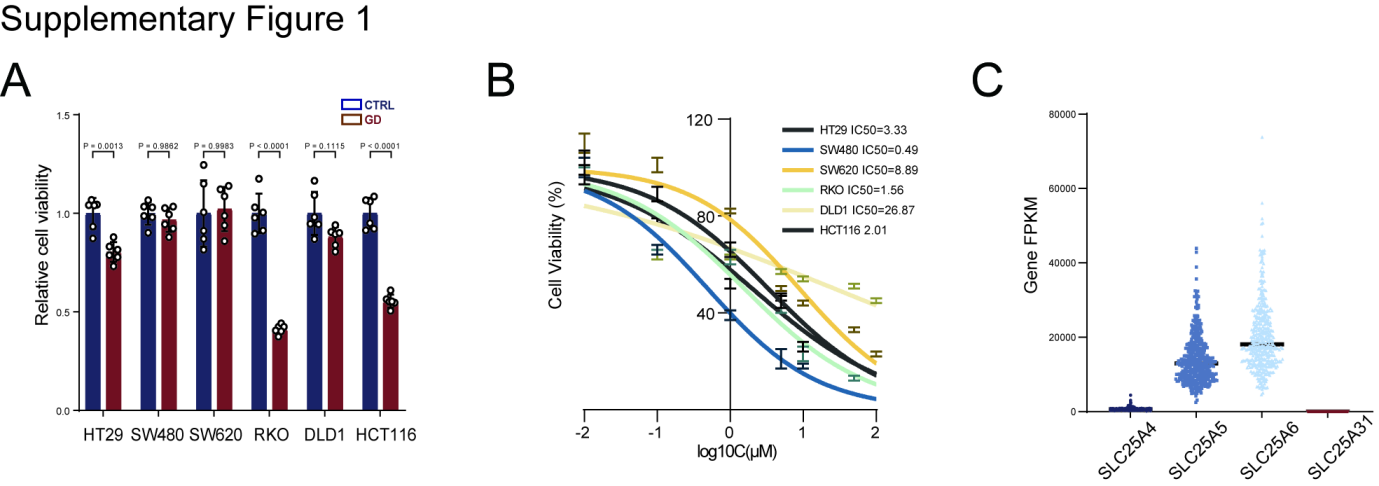
Supplementary data**

**Supplementary Figure 1. Screening of CRC cells for GD sensitivity.**

(A, B) Viability of CRC cell lines after glutamine deprivation or CB-839 treatment. (C) Analysis of TCGA COAD and READ datasets showing relative levels of SLC25A4, SLC25A5, SLC25A6, and SLC25A31. Data are presented as the mean ± SD (*n* = 3). Error bars represent the SD. Exact *P*-values are shown.


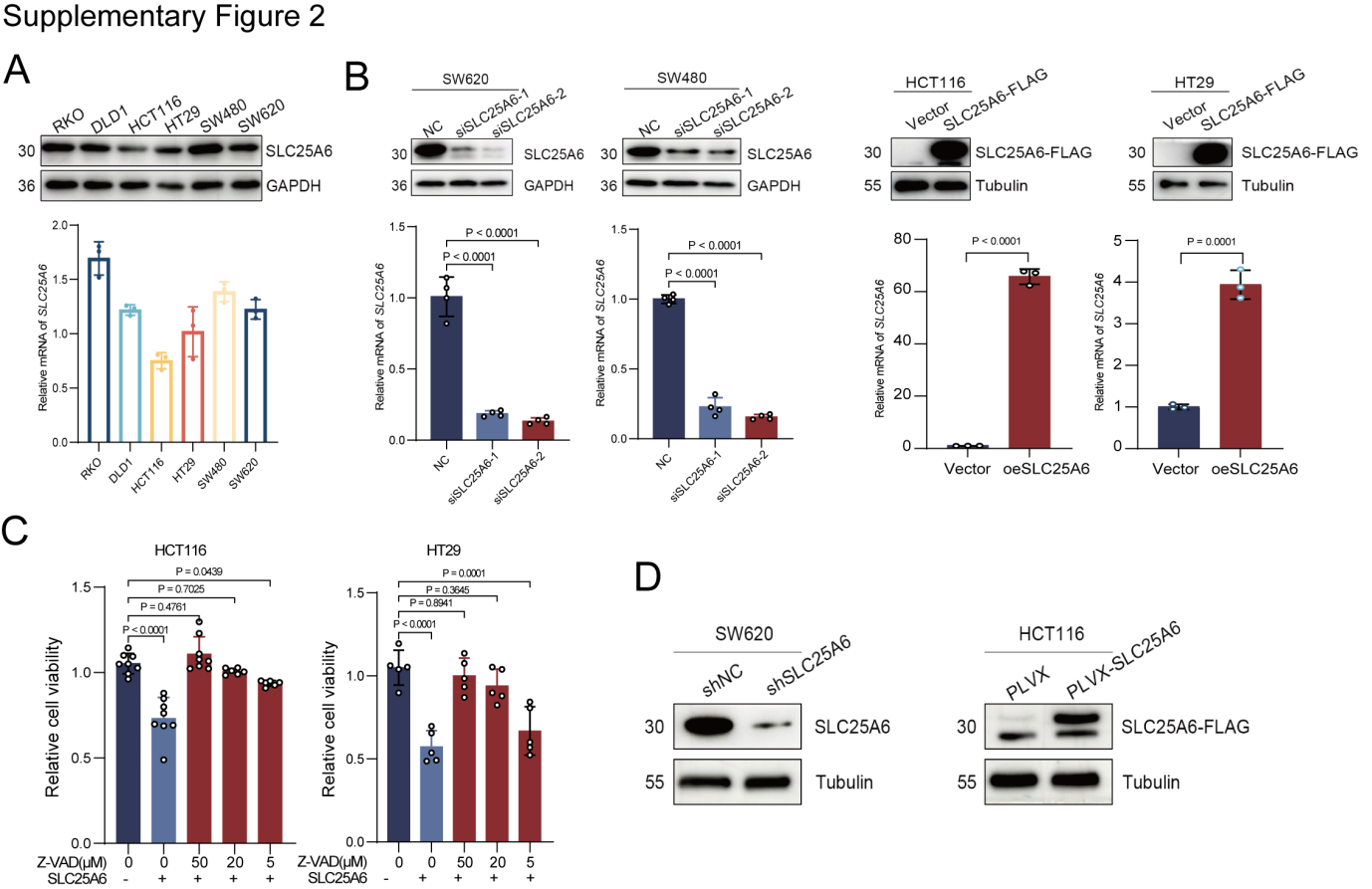
**Supplementary Figure 2. Validation of SLC25A6 manipulation and apoptosis assays.**

(A) qPCR and representative immunoblotting images of SLC25A6 expression in CRC cell lines. (B) qPCR and representative images of immunoblots validating transient SLC25A6 overexpression and knockdown. (C) Cell viability assay of SLC25A6-OE cells with increasing concentrations of Z-VAD. (D) Representative images of immunoblots for SLC25A6 expression in stably transfected control, overexpression, and knockdown CRC cells. Data are presented as the mean ± SD (*n* = 3). Error bars represent the SD. Exact *P*-values are indicated.


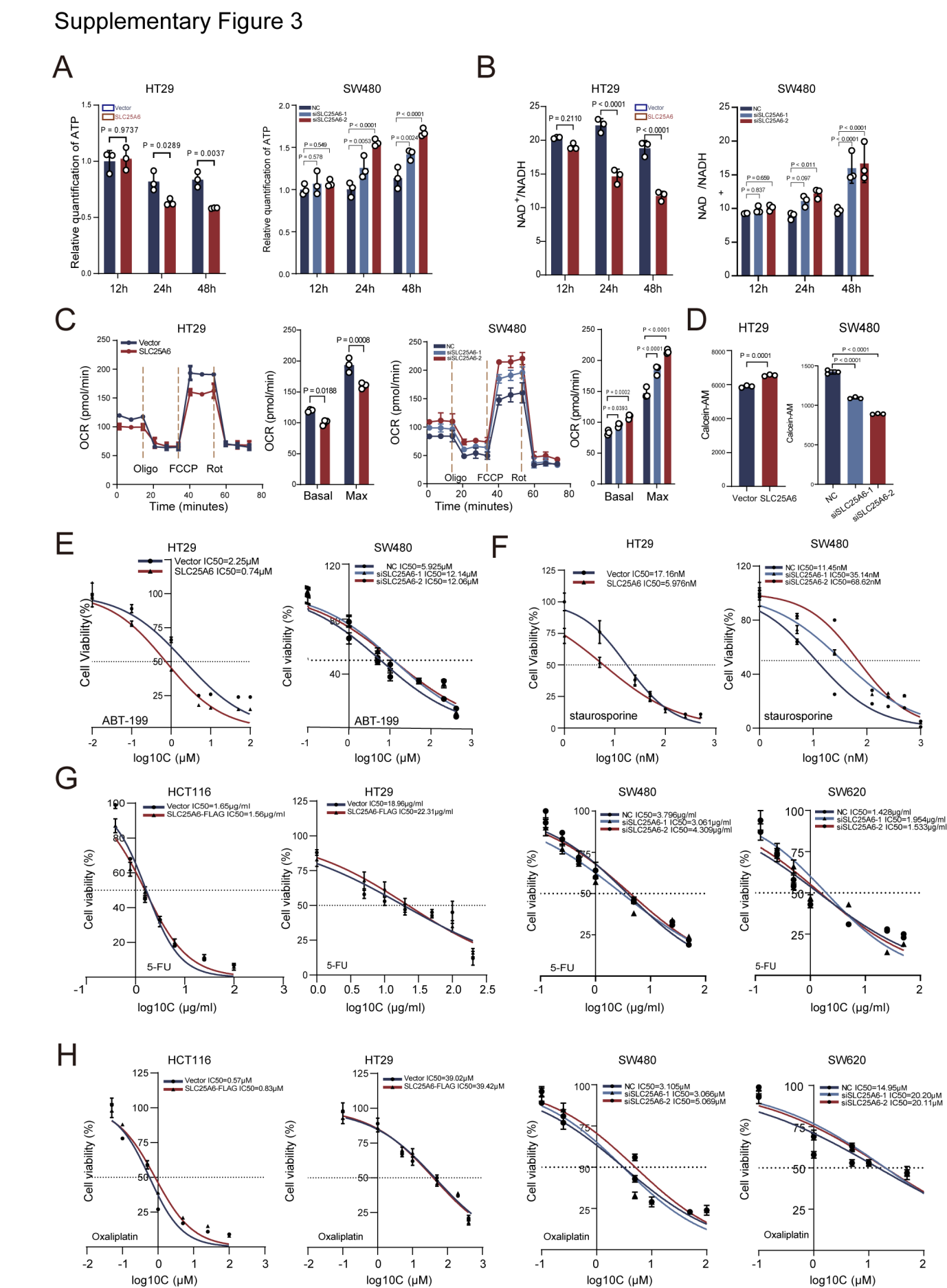


**Supplementary Figure 3. Drug sensitivity in SLC25A6-overexpressing CRC cells.**

(A) Quantification of cellular ATP levels. (B) Quantification of NAD⁺/NADH ratio. (C) Oxygen consumption rate measured using Seahorse assay. (D) Quantification of mPTP opening. (E–H) Cell viability assays following treatment with (E) ABT-199, (F) staurosporine, (G) 5-fluorouracil or (H)oxaliplatin. Data are presented as the mean ± SD (*n* = 3). Error bars represent the SD. Exact *P*-values are indicated.


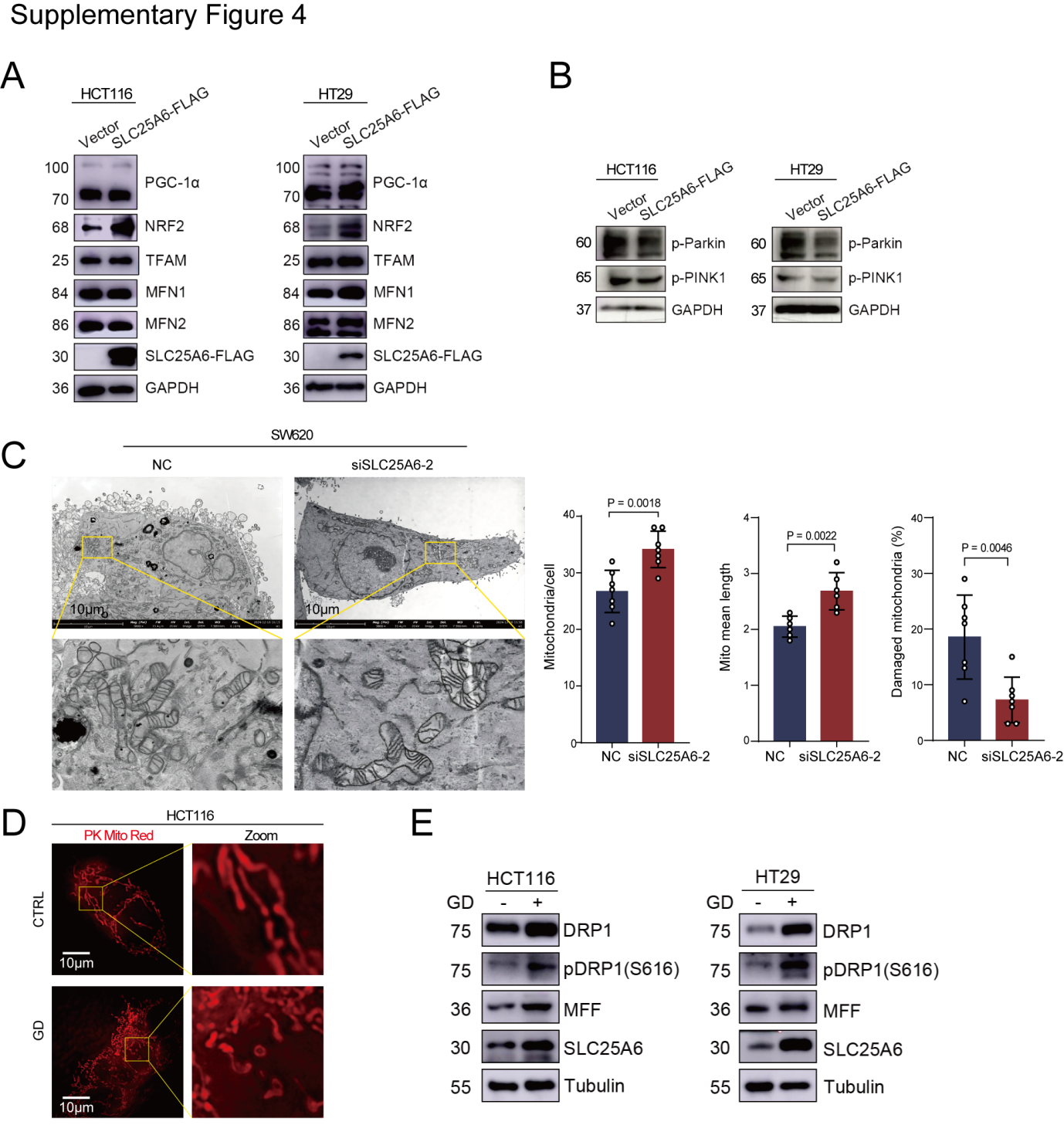


**Supplementary Figure 4. Effects of SLC25A6 on mitochondrial integrity, biogenesis, and mitophagy.**

(A) Immunoblotting of mitochondrial fusion proteins (MFN1, MFN2) and biogenesis-related proteins (PGC1α, TFAM). (B) Immunoblotting of phosphorylated PINK1 and Parkin in control and SLC25A6-OE cells. (C) Transmission electron microscopy of mitochondria in SLC25A6-KD cells. (D) Representative images of immunofluorescence analysis of mitochondrial morphology in CRC cells under GD (scale bar, 5 μm). (E) Representative images of immunoblots for mitochondrial fission proteins in CRC cells cultured under GD. Data are presented as the mean ± SD (*n* = 3). Error bars represent the SD. Exact *P*-values are indicated.


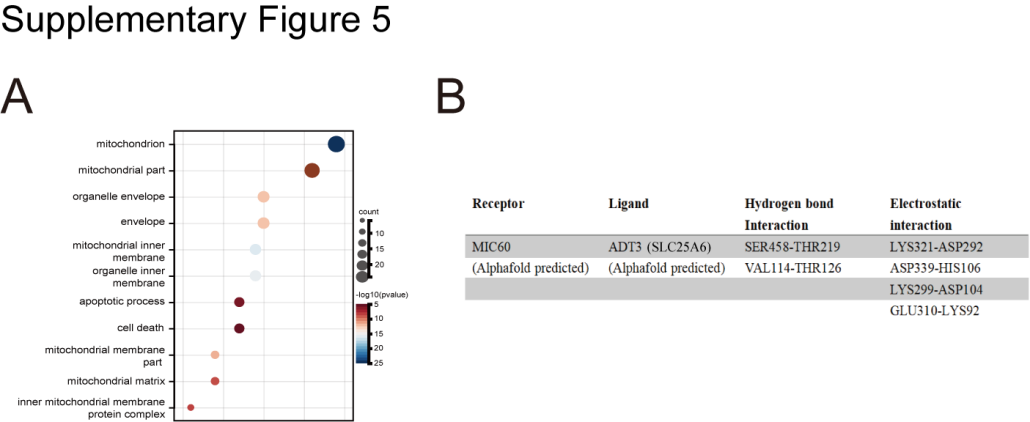


**Supplementary Figure 5. Identification of SLC25A6-associated proteins and potential binding sites.**

(A) Bubble plot analysis of SLC25A6 immunoprecipitation–mass spectrometry results. (B) List of predicted interaction sites from molecular docking analysis.

**
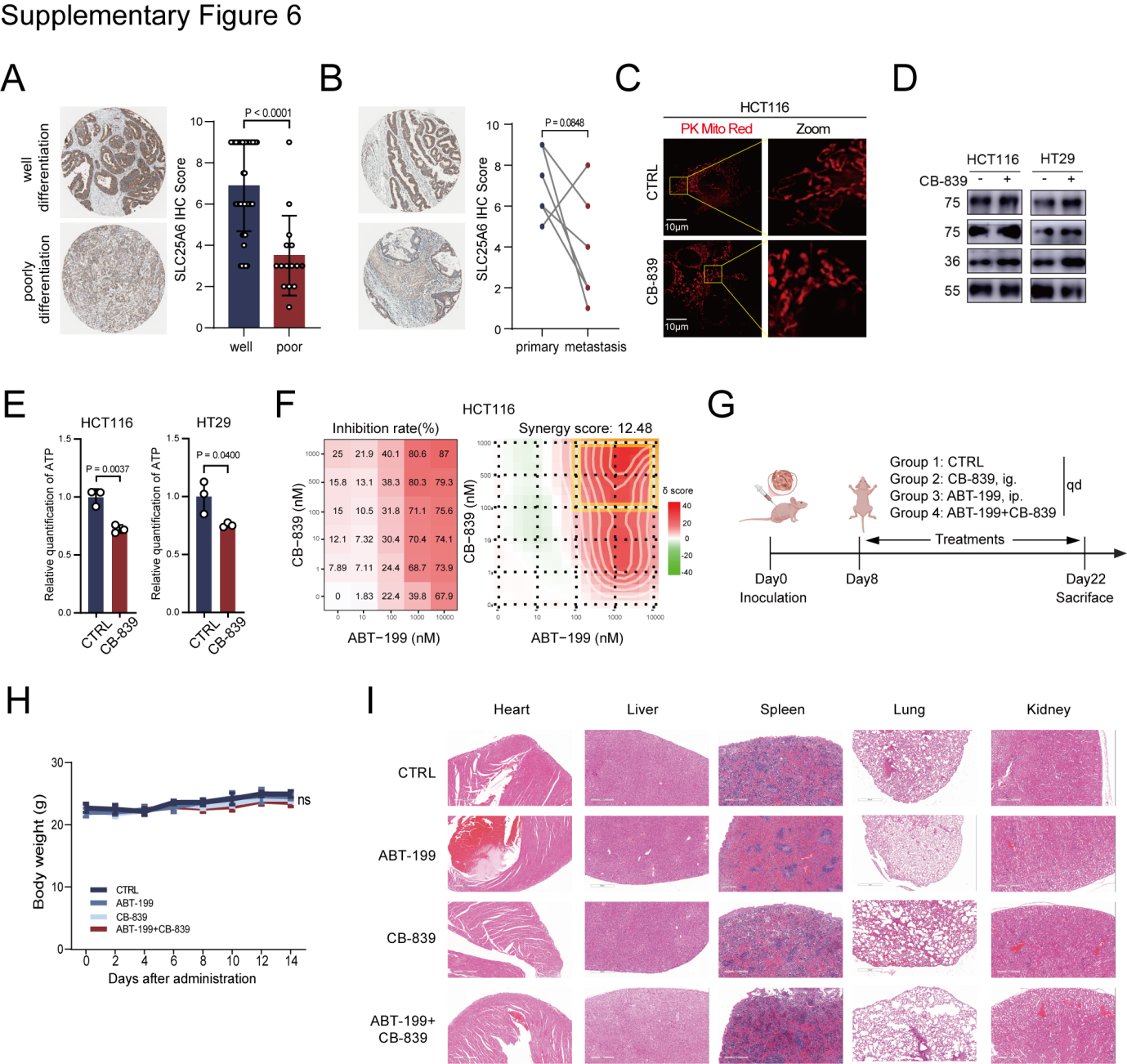
**

**Supplementary Figure 6. SLC25A6 expression in CRC samples and safety assessment of combination therapy.**

1. Comparison of SLC25A6 expression between well-differentiated (*n* = 15) and poorly differentiated (*n* = 47) CRC tumors. (B) Comparison of SLC25A6 expression between paired primary tumors and metastatic tumors (*n* = 6) CRC tumors. (C) Representative immunofluorescence staining images of mitochondrial morphology in control and CB-839 treated cells (scale bar: 10 μm). (D) Immunoblot analysis of mitochondrial fission-related proteins. (E) Quantification of cellular ATP levels. (F) Analysis of CB-839 and ABT-199 synergy in CRC cell line HCT116. (G) Schematic illustration of the animal experimental workflow. (H) Body weight monitoring of mice treated with vehicle, CB-839, ABT-199, or combination treatment. (I) Hematoxylin and eosin staining of major organs (heart, liver, spleen, lung, and kidney) from xenograft-bearing mice after treatment (scale bar: 400 μm). Data are presented as the mean ± SD (*n* = 3). n.s., non-significant. Error bars represent the SD. Exact *P*-values are indicated.

**Supplementary Table 1. Clinical characteristics of patients with colorectal cancer (CRC).**

**Supplementary Table 2. siRNA sequences targeting SLC25A6.**

**Supplementary Table 3. Primer sequences used for quantitative real-time PCR (qPCR).**

**Supplementary Table 4. Antibodies used in immunohistochemistry (IHC) and Western blotting.**
