# Supplementary figures and images for: Glutamine metabolic stress induces SLC25A6-dependent mitofission via MIC60–MIC19 complex disassembly in colorectal cancer
Source: Cell Death Dis. 2026 Apr 23;17(1):537. doi: 10.1038/s41419-026-08754-6 (PMC13237379; doi:10.1038/s41419-026-08754-6)

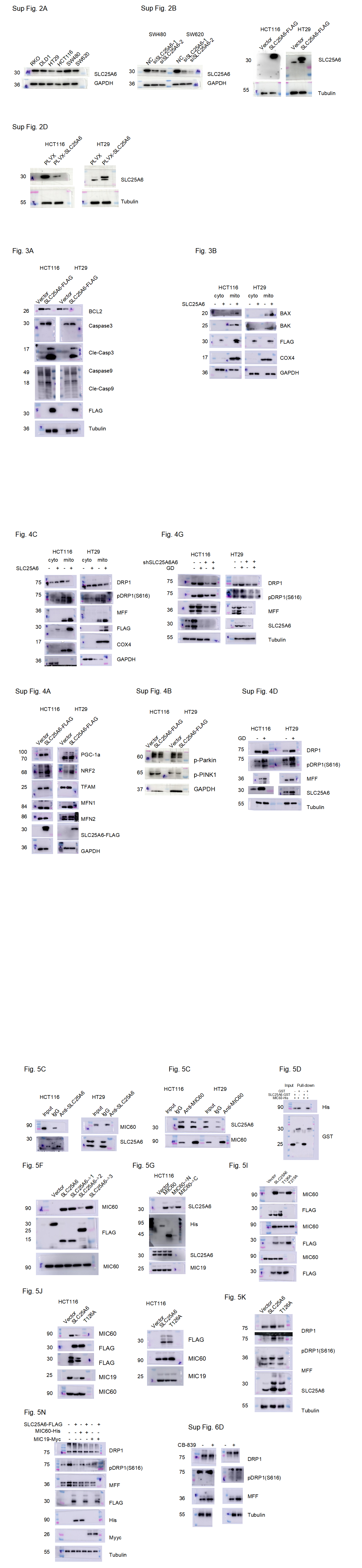

Supplement: Supplementary file 2 — original data [file 41419_2026_8754_MOESM2_ESM.tif]
